# Evaluating refrigeration and antibiotic treatment for maintaining urine electrophysiology

**DOI:** 10.1371/journal.pone.0319089

**Published:** 2025-02-25

**Authors:** Rashedul Hoque, Fatima H. Labeed, Srdjan Cirovic, Michael Pycraft Hughes

**Affiliations:** 1 Centre for Biomedical Engineering, School of Engineering, University of Surrey, Guildford, United Kingdom; 2 Department of Biology, UAEU University, Al Ain, United Arab Emirates; 3 Department of Biomedical Engineering and Biotechnology/Healthcare Engineering Innovation Group, Khalifa University of Science and Technology, Abu Dhabi, United Arab Emirates; Bowen University, NIGERIA

## Abstract

**Background:**

Electrophysiological analysis of urine has shown utility in differentiating between healthy and bladder cancer specimens, offering a rapid, label-free alternative to molecular methods. However, transporting and preserving urine samples from collection to the laboratory poses logistical challenges that could impact the reliability of electrophysiological measurements.

**Objective:**

This study investigates the effects of prolonged refrigeration on the dielectric properties and ζ-potential of urine specimens and evaluate whether antibiotic treatment can enhance sample preservation without altering electrophysiological properties. A new methodology to evaluate urine specimen quality and determine bacterial contamination, using electrophysiological modalities, is presented.

**Methods:**

Mid-stream urine samples from healthy participants (n =  4) were collected and divided into untreated and 1% penicillin/streptomycin-treated groups. Samples were analysed at baseline prior to storage at 4°C, with further analysis every 24 hours for 96 hours. Changes in dielectrophoresis (DEP) response and ζ-potential were measured using a 3DEP cytometer (Deparator, UK) and Malvern Panalytical Zetasizer Nano ZS90 (Malvern, UK), respectively. Chemical analyses, including pH and nitrite levels, and microscopic examinations were also conducted.

**Results & Limitations:**

Significant electrophysiological changes were observed in both untreated and antibiotic-treated urine samples over time. Both groups showed a linear increase of change in DEP response and ζ-potential values, from baseline over time. Untreated samples exhibited significant deviations in DEP and ζ-potential from baseline after 48 hours, with significance at 72 hours (P < 0.05). Treated samples only showed significant changes in ζ-potential at 96 hours (P < 0.05). Chemical analysis indicated increased pH and nitrite presence in untreated samples at 48 hours, indicating bacterial growth. Treated samples took more than 48 hours to show changes in both chemical parameters. Limitations include the relatively small study sample size, not evaluating the preservatory effects of UTI-specific antibiotics, such as nitrofurantoin and trimethoprim, and exploring different drug concentrations.

**Conclusion:**

Prolonged refrigeration can maintain the quality of urine samples for up to 48 hours with antibiotic treatment. Current UK and European guidelines recommend urinalysis within 24 hours of specimen collection; the findings of this study support the use of DEP and ζ-potential analysis as practical clinical tests in a mail-in screening setting, provided appropriate sample preservation measures are taken.

## 1 Introduction

Analysis of urine is a vital, non-invasive and cost-effective method in clinical practice to help detect and monitor numerous conditions, including bladder cancer. However, the transportation of clinical urine samples from clinic to laboratory, while maximising sample preservation and mitigating contamination risks, presents a considerable logistical challenge. In principle, a timely sample shipment would mean clinic to laboratory within 24 hours of collection; however, unexpected delays in shipping are not uncommon, which may lead sample receipt beyond the ideal timeframe. Within clinical pathology laboratories, the process of analysing samples is divided into three distinct phases: pre-analytical, analytical, and post-analytical. The pre-analytical phase, which includes sample collection, processing, storage and transport, is essential for ensuring the quality and efficiency of the biomedical laboratory services [1]. Between 46% and 70% of laboratory errors occur throughout the pre-analytical phase [2,3], risking erroneous results that adversely impact clinical diagnoses and patient healthcare. Therefore, urine transport and storage should follow a procedure aiming to obtain a sample that closely matches the cellular, physiochemical and microbial properties at time of micturition, during the analytical phase.

Cellular components in urine are relatively easy to access, offering key diagnostic information. Erythrocytes can signal urinary tract infections (UTIs) or cancer, with abnormal shapes indicating renal issues [4]. Squamous epithelial cells may suggest poor sample collection if abundant, while high numbers of transitional epithelial cells indicate urinary tract inflammation. Rare deep urothelial cells can point to injury or chronic infection, and elevated leukocytes often show inflammation or infection [4]. The quality of a urine sample is closely linked to its clinical value, as improper storage conditions post-collection can significantly alter its composition, compromising the accuracy of diagnostic results.

Ribeiro et al. [5] undertook the sediment analysis of 80 urine samples stored for 24 h at room temperature and in 2–8°C refrigeration. Samples stored at room temperature showed an elevation in pH, the presence of nitrite, and a reduction in leukocytes and glucose. The increase in nitrite and decrease in glucose was associated with bacterial metabolism; in samples that were free from bacterial contamination, 17.5% of those maintained at room temperature showed substantial bacteriuria after 24 hours. Conversely, refrigerated samples exhibited no alterations in cell counts when compared to the initial examination at the time of collection, even after a period exceeding 24 hours. Refrigeration was not observed to disrupt chemical tests, maintained cell structure, and inhibited bacterial proliferation [5], underscoring the need of chilling for preserving urine. Studies have reported the detrimental changes in unpreserved urine specimens after 2 hours at room temperature, suggesting that analysis should be completed within this limited timeframe [5–8]. Standards for microbiology investigations [9] state that if specimens cannot be transported and processed within 4 hours, and if processing is delayed for up to 48 hours, then refrigeration is “essential”. Ahmed and Tom [10] studied the effect of delayed fixation of urine specimens on urine cell preservation. Examining 50 patient urine samples processed via multiple methodologies, they found that of those processed immediately, 94% were “well preserved” and only 6% were “partially degenerated”. In contrast, the specimens stored at room temperature for 2 hours had 50% of observed cells considered “partially degenerated” and 22% “completely degenerated.” The percentage of “partially degenerated” cells remained the same in samples processed after 4-hour storage at room temperature, but 40% showed “completely degenerated” cells. The researchers concluded that pH and duration between urine collection and preparation affects cell morphology, leading to swelling and loss of internal structure, and increases risks of bacterial and fungal contamination.

Whilst these studies show the susceptibility of urine solutions for conventional chemical analyses, they do not describe the effect of storage conditions on the electrophysiological properties of such samples which are of growing interest for diagnosis and monitoring [11–13]. For example, dielectrophoresis (DEP) is a technique that has gained particular attention in the field of biomedicine due to its ability to characterise and separate cells based on their distinct dielectric phenotype [11]. DEP occurs when a polarizable particle, such as a cell, is exposed to a non-uniform electric field. The cell migrates either towards or away from regions of high electric field gradient depending on the cell properties at the applied frequency; the direction and magnitude of motion are influenced by the cell’s electrophysiological properties, determined by factors including cell shape, size and membrane surface characteristics, as well as the frequency of the applied voltage [14]. Analysis of the DEP response of cells across a spectrum of frequencies allows measurement of their electrophysiological properties[15]. As DEP does not require the use of antibodies or stains for cell labelling, it serves as a highly efficient tool for the rapid assessment of cell properties, particularly in scenarios where biomarkers are unknown or inconsistently expressed. DEP has been used for analysis of circulating tumour cells [16,17], bacteria [18,19] and viruses [20,21], and characterising the effects of different drug treatments [22,23], as well as a range of cancers including colon, breast, prostate and ovarian cancer [17,24–26]. In the clinical setting, Hughes et al. [27] conducted a prospective proof-of-concept study to distinguish between oral squamous cell carcinoma from normal oral epithelium based on the dielectric properties, using a commercially available 3DEP cytometer (Deparator, UK). The system was able to discern oral squamous cell carcinoma, yielding a sensitivity and specificity of 92.0% and 94.5%, respectively. Building on this, Hoque et al. [28] undertook a pilot study (n =  16) to determine the DEP response of the solid constituents (pellets) of voided urine specimens from bladder cancer patients and healthy participants using a novel analysis parameter, [27,29]. Urine specimens from bladder cancer patients were distinguished from healthy controls at a sensitivity of 75% and specificity of 88% [28]; when low-cellularity samples were excluded, sensitivity increased to 100% (specificity was unaffected). Samples were analysed within 20 hours of collection, suggesting that DEP analysis could be adapted for mail-in or centralized collection services, allowing a single 3DEP device to process samples for disease screening from a wide geographic area.

Another electrophysiological phenomenon that can be measured is the cellular zeta (ζ)-potential. This is the electrical potential a few nanometers (nm) outside the cell surface, which affects how the cell interacts with its environment. It is a measure of the charge distribution on the cell’s surface, indicating the level of electrostatic attraction or repulsion, and plays a crucial role in processes such as cell aggregation and cell-particle interactions [30,31]. Although applications of ζ-potential analysis is in relatively early stages within biomedicine, studies have demonstrated its capability to record cell-particle interactions, including with drug-delivery vectors [32], and its usage as a potential label-free marker for apoptosis and reduced cell viability [33]. Both the membrane potential and ζ-potential of breast cancer cell lines were discovered to be significantly depolarised compared to their healthy counterparts, highlighting ζ-potential as a potential disease biomarker [32].

In this paper, we build upon the findings of Hoque et al. [28] by investigating whether electrophysiological analysis of urine specimens could still be used as an effective screening or diagnostic tool, while facing the logistical challenges associated with clinical testing. The effects of prolonged urine refrigeration on the electrophysiology of specimens (DEP and ζ-potential) were determined. Additional investigations were undertaken as to whether antibiotic treatment of urine specimens, prior to storage in refrigeration, prolonged sample preservation compared to standard untreated urine specimens.

## 2 Materials and methods

### 2.1 Participant recruitment

This study received a favourable ethical opinion by the University of Surrey ethics committee (FEPS 20-21 023 EGA), and samples were collected and processed following UK and European Federation of Clinical Chemistry and Laboratory Medicine (EFLM) urinalysis guidelines [9,34]. The inclusion criteria included participants at least 18 years old, who have not been diagnosed and receiving treatment for any urological conditions and generally consider themselves healthy. All participants received an information sheet and provided verbal and written informed consent prior to providing a urine specimen. A total of 4 participants provided morning, mid-stream voided urine specimens. Good Clinical Practice [35] was adhered to throughout the study and samples were collected, processed and analysed in accordance with the Human Tissue Act 2004 [36]. Participant personal information was anonymised by designating each participant with a unique identification number.

### 2.2 Sample processing

**Fig 1:**
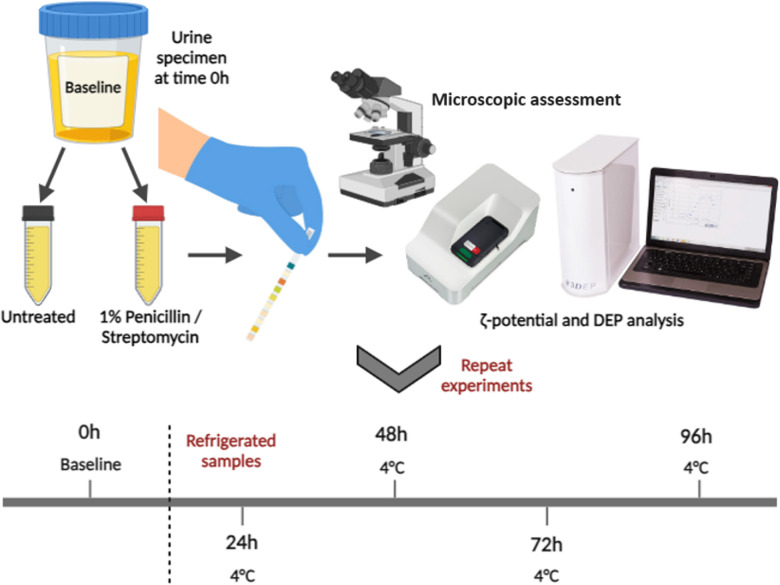
Experimental process to determine the effects of prolonged urine refrigeration at 4°C on healthy urine specimen electrophysiology, and whether 1% penicillin/streptomycin treatment effects sample electrophysiology and extends preservation. ζ-potential and the MDV were the electrophysiological analysis parameters assessed, analysed via the Malvern Panalytical Zetasizer Nano ZS90 and the 3DEP cytometer respectively. Changes in pH and nitrite testing were the most relevant chemical analyses, exhibiting changes over time due to bacterial growth. Created with Biorender.com.

A diagram of the experimental process is displayed in Fig 1. Participants provided ~ 50 mL of urine. Macroscopic assessment of urine specimens (colour, odour, turbidity) was undertaken prior to analysis. Samples were then analysed immediately via a standard dipstick reagent strip (ONE+STEP^®^, South Korea) to detect leukocytes, nitrite, protein, glucose, ketones, urobilinogen, bilirubin, blood and pH. All samples were considered to be uninfected at time of collection. Each sample was split into two vials, one treated with 1% penicillin/streptomycin (Sigma-Aldrich, USA) and the other left untreated. 20% of each sample was used for electrophysiological analysis, with the original two sample containers stored in refrigeration at 4°C.

An isosmotic medium containing 248 mM sucrose, 16.7 mM dextrose, 250 μM MgCl_2_ and 100 μM CaCl_2_, was prepared, with osmolarity determined via the Osmomat 3000 (Gonotec, US) and adjusted to 290–299 mOsm. Conductivity of the final DEP medium was corrected to 43 mS.m^−1^ by adding phosphate buffered saline, verified using a Jenway 470 conductivity meter (Bibby Scientific). Both vials of untreated and treated samples were evenly split so that each group had 2 vials, one for DEP analysis and one for ζ-potential analysis. All sample containers underwent centrifugation at 1000 G for 10 minutes. The supernatant was aspirated in Virkon^®^ disinfectant solution and the pellet resuspended in fresh DEP medium, followed by another centrifugation step for 5 minutes. Once the supernatant was disposed of again, the samples were resuspended in the same DEP medium, at 1 mL for DEP analysis and 800 µL for ζ-potential analysis, for both the untreated and 1% penicillin/streptomycin treated groups.

### 2.3 DEP analysis and ζ-potential analysis

DEP analysis was performed using  a 3DEP cytometer, where approximately 5 µL of resuspended urine specimen was injected into a disposable 20 well DEP chip which was then loaded into the 3DEP reader. Samples were analysed at frequency ranges between 10 kHz and 45 MHz at 10V peak-to-peak for 30 seconds. A minimum of three technical repeats were undertaken and the resultant DEP spectra were exported to Excel (Microsoft, Redmond WA) for analysis. The established mean difference value (MDV) index test was determined from the averaged DEP spectra of each participant, as per previously published protocols [27–29,37]. The Malvern Panalytical Zetasizer Nano ZS90 (Malvern, UK) was used to measure ζ-potential. 800 µL of specimen solution was injected into a disposable cuvette for ζ-potential analysis. 10 µL of sample solution was loaded into a C-Chip haemocytometer for microscopic visualisation. The experimental procedure undertaken at time of collection (0 h or baseline), was repeated every 24 hours (24 h, 48 h, 72 h and 96 h) to determine changes in sample electrophysiology (MDV and ζ-potential) and bacterial growth.

### 2.4 Statistical analysis

All statistical analysis was undertaken via Microsoft Excel and GraphPad Prism 9 (Dotmatics, USA). To normalise the MDV and ζ-potential data, the analysis compared the average percentage changes in the untreated and treated groups over time from baseline (time 0-hour). As all urine samples were screened to be healthy, cleared of UTIs at the time of collection, most of the chemical parameters associated with transient infections or urological disease were negative or normal throughout the entire 96-hour testing duration. These were the leukocytes, protein, glucose, ketones, urobilinogen, bilirubin and blood parameters. The parameters that were anticipated to change over prolonged storage were nitrite and pH, which suggest the presence of bacteria. Nitrites are typically absent in urine but can form when bacteria convert urinary nitrates to nitrites [38]. All samples were confirmed to test negative for nitrites at baseline, with the absence of bacterial colonies confirmed via microscopy. Nitrite results are presented on an ordinal scale of negative, positive (≥0.05 mg/dL) and trace (detectable but below 0.05 mg/dL minimum sensitivity threshold) [39]. A variety of statistical tests were performed. An Analysis of Variance (ANOVA) test was undertaken to determine any significant differences in the electrophysiological and chemical test results over time. Tukey’s Honest Significant Difference (HSD) post hoc test to analyse pairwise differences. A t-test was also performed to determine significance between the results of each timepoint to baseline values. Statistical significance was observed when *P ≤ 0.05*.

## 3 Results

**Fig 2:**
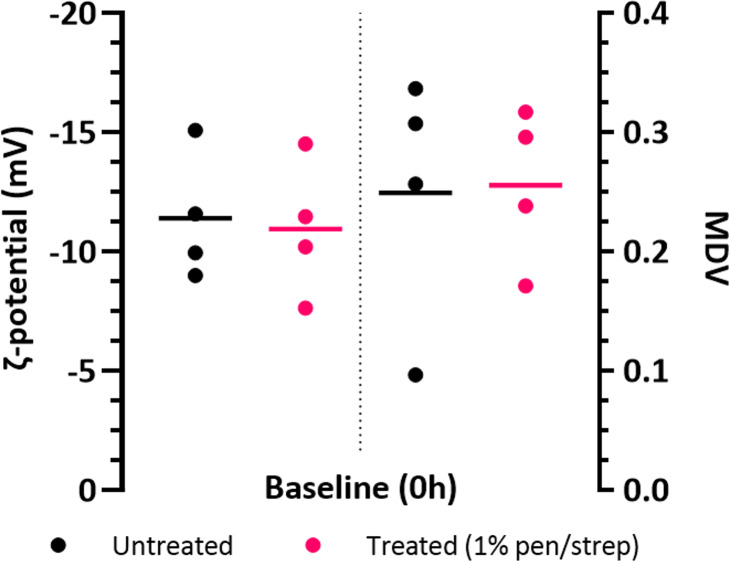
ζ-potential and MDV of voided urine samples (with mean), analysed immediately after collection (baseline), for both untreated urine and with 1% penicillin/streptomycin treatment. There was no significant difference in both the ζ-potential and MDVs of each study group (P > 0.05).

**Fig 3:**
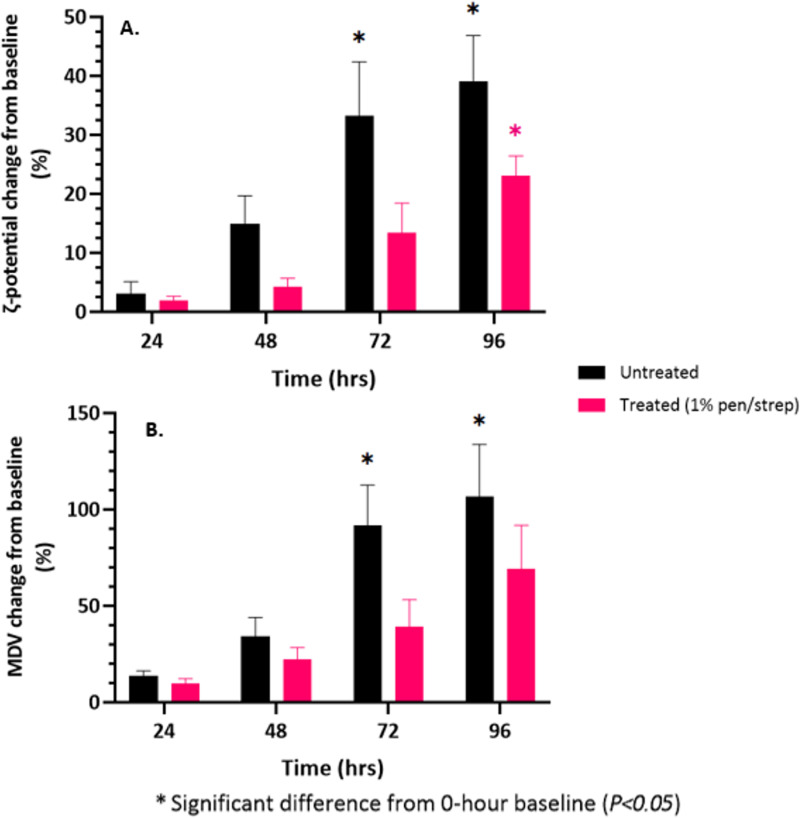
The impact of the 1% penicillin/streptomycin treatment on urine preservation is shown by the percentage change from baseline (0-hour) with SEM in ζ-potential (A) and MDV (B) of healthy urine stored at 4°C for 96 hours. Untreated urine (black) exhibited significant changes in ζ-potential at 72 hours (P =  0.035) and 96 hours (P =  0.015), and in MDV at 72 hours (P =  0.021) and 96 hours (P =  0.028). In contrast, the 1% penicillin/streptomycin treated group (pink) showed significant changes in ζ-potential only at 96 hours (P =  0.01) and no significant change in MDV, suggesting improved preservation for electrophysiological analysis with the treatment method.

The results of the DEP data (MDV) and ζ-potential analysis showed noticeable changes in the parameters over time in both the untreated and 1% penicillin/streptomycin treated groups. The MDV and ζ-potential data of each participant sample at time 0h are shown in Fig 2, and changes observed from baseline (time 0-hour) shown in Fig 3.

Considerable electrophysiological changes were observed in urine samples following prolonged storage. In the untreated group, both the MDV and ζ-potential appeared to deviate from the 0-hour baseline from 48 hours onwards; deviations from baseline were observed in the antibiotic group from 72 hours onwards. There was a trend observed in the untreated group where the ζ-potential lowered over time. This was not a consistent trend in the treated group. Aside from clear deviations from baseline values, there was no pattern as to whether the MDV in the treated group would increase or decrease over time. Conversely, the MDV in the antibiotic treated group noticeably increased with prolonged storage in refrigeration.

As the study was interested in whether 1% penicillin/streptomycin treatment of voided urine has a preservative effect on specimens, compared to untreated urine, it is important to determine whether treatment maintains the same electrophysiological characteristics as untreated urine or causes alterations. Fig 2 shows the individual ζ-potential and MDVs of urine specimens, both untreated and with the antibiotic treatment, at baseline The mean ζ-potential was −11.40 ±  2.63 mV (mean ±  standard deviation) for the baseline untreated group and −10.94 ±  2.86 mV for the treated group; the median was −10.76 mV and −10.82 mV, respectively. There was no significant difference in the ζ-potential and MDVs of the urine specimens between the untreated and treated groups at baseline (*P > 0.05*), suggesting acute 1% penicillin/streptomycin treatment does not affect the electrophysiology of samples.

To account for the variation in baseline values of the MDV and ζ-potential of each participant sample, the percentage change from baseline values over time was used to compare both study groups. As the untreated and antibiotic treated groups showed no significant difference at baseline (Fig 2), the magnitude of percentage deviation from baseline over the course of 96 hours in refrigerated storage can be compared between study groups.

The percentage change from baseline for the untreated and treated study groups is shown in Fig 3A and Fig 3B, for ζ-potential and MDV respectively. In general, both the untreated and treated groups show negligible changes in ζ-potential and MDV from baseline after 24 hours of storage in refrigeration. A consistent pattern of increase is observable from 48 hours onwards for the remainder of the study. The one-way ANOVA showed there was a statistically significant difference in the percentage change from baseline in ζ-potential between timepoints for both the untreated group (F (3, 12) =  6.524, *P =  0.007*) and the treated group (F (3, 12) =  9.644, *P =  0.002*). In the untreated group, Tukey’s Honestly Significant Difference (HSD) test found the difference in percentage change was significant between 24 hours versus 72 hours and 24 hours versus 96 hours (*P < 0.05* for both); the percentage change from baseline was significantly different at 96 hours in the treated group, when compared to 24 hours and 48 hours (*P < 0.05* for both).

A similar trend was observed in the percentage change in MDV from baseline. Interestingly, the one-way ANOVA showed there was a statistically significant difference from baseline in MDV between timepoints for the untreated group (F (3, 12) =  6, *P =  0.008*) but not the treated group (*P > 0.05*). Tukey’s HSD identified a significant difference in the percentage change in MDV between 24 hours versus 72 hours and 24 hours verses 96 hours in the untreated group (P < 0.05 for both). There was only a significant difference in values between the 24-hour and 96-hour timepoints in the treated group (*P < 0.05)*.

**Fig 4:**
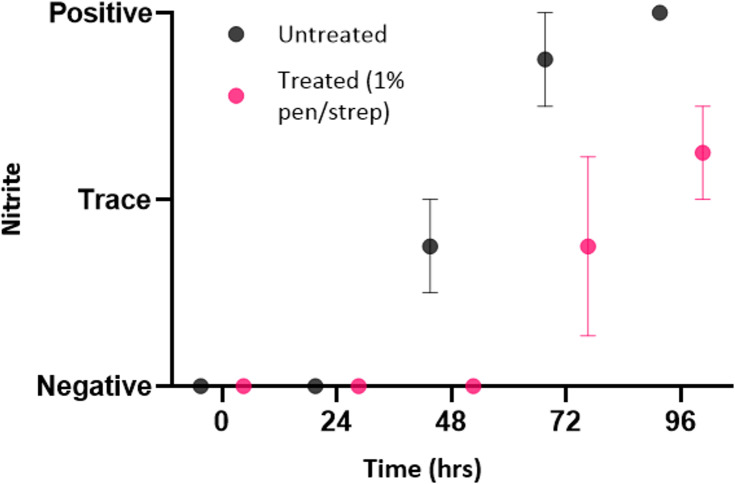
Dipstick reagent strip results for urinary nitrites (mean±  SEM) for specimens stored in refrigeration over time, either untreated or with 1% penicillin/ streptomycin treatment.

The chemical analysis results identified trends in both groups. As shown in Fig 4, while both the untreated and treated groups reported negative nitrite from 0–24 hours, trace levels of nitrite were detected in the untreated sample group at 48 hours; 75% of untreated samples were positive after 72 hours and all samples tested positive for nitrite at 96 hours. The one-way ANOVA showed there was a statistically significant difference from baseline in pH between timepoints for the untreated group (F (1.699, 5.098) =  10.78, *P =  0.016*). Tukey’s HSD identified a significant difference in the percentage change in pH between baseline and 96 hours and 24 hours versus 96 hours (*P < 0.05* for both). Conversley, all samples in the 1% penicillin/streptomycin treatment group tested negative for nitrite from 0–48 hours; 50% of samples tested either trace or positive at 72 hours and all samples tested positive at 96 hours. The one-way ANOVA showed there was a statistically significant difference from baseline in pH between timepoints for the treated group (F (1, 3) =  25, *P =  0.015*). Tukey’s HSD did not identify significance, suggesting that while there is an overall difference in means among the timepoints, the difference is not attributable to any specific pairwise comparisons. The presence of nitrite in samples after prolonged storage in refrigeration, indicated the bacteriostatic effects of refrigeration are limited to 24 hours in untreated raw specimens and up to 48 hours in samples treated with 1% penicillin/streptomycin.

**Fig 5:**
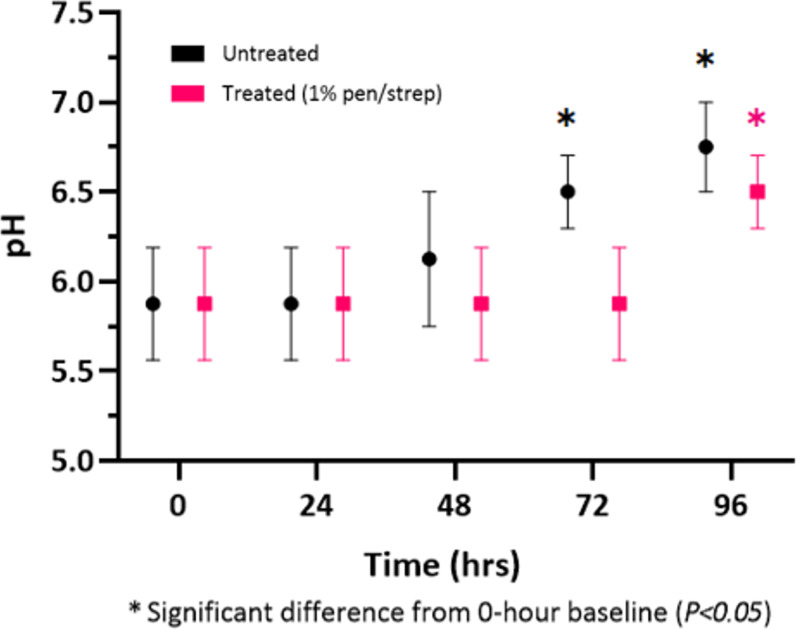
Dipstick reagent strip results for changes in urinary pH (mean±  SEM) for specimens stored in refrigeration over time, either untreated or with 1% penicillin/streptomycin treatment.

The pH of samples (Fig 5) exhibited a similar trend to the nitrite findings, with both groups exhibiting no change from baseline for up to 24 hours. The untreated group then showed a linear increase in pH from 48 hours onwards, with a significant increase compared to baseline being observed at 72 and 96 hours (*P < 0.05* for both). In contrast, the urinary pH in the antibiotic treated group remained constant until 96 hours when a significant increase was observed (*P < 0.05*). This suggests a delayed onset of bacterial growth in samples treated with 1% penicillin/streptomycin, minimising electrophysiological changes in treated specimens for at least 48 hours, as opposed to the 24 hour preservation observed in untreated samples.

## 4 Discussion

Our results suggest that refrigerating at 4°C alone does preserve the electrophysiological properties of urine specimens for at least 24 hours. There was both no sign of bacterial growth microscopically at 24 hours and chemical analysis did not identify the presence of nitrites. However, trace levels of nitrites were detected at 48 hours, with positive nitrite tests determined at 72 and 96 hours. It is worth noting, while the dipstick reagent tests used in the study possessed a “trace” scaling for nitrites, most dipstick tests only have negative or positive testing, with the current NICE guidelines employing the binary test scale [40]. Therefore, a “trace” recording of nitrites would likely be considered a positive test result by clinicians and pathologists. Furthermore, there was a notable increase in pH at 48 hours for untreated from baseline, with pH at 72 and 96 hours increasing significantly from baseline (*P < 0.05*). Although urinary pH can range from 4.5 to 8, it is normally slightly acidic (5.5–6.5), due to metabolic activity, with alkali urine associated with urease splitting bacteria [38]. An increase in urinary pH over time, alongside positive nitrite testing, suggested the presence of bacteria in urine samples after prolonged refrigeration beyond 24 hours. Our findings also suggest that refrigerating urine specimens at 4°C with a 1% treatment of penicillin/streptomycin extends sample preservation. Elevation in pH and trace/ positive nitrite testing were observed at 72 hours, suggesting that antibiotic treatment extended sample preservation by another 24 hours (48 hours post baseline timepoint) compared to the untreated group. This combined method of antibiotic treatment and refrigeration offers clinical scientists a significant improvement in preservation over standard practice, where EFLM guidelines state that samples refrigerated alone “should” be examined within a strict 24-hour timeframe [34]. It was also identified that urine samples, both untreated and antibiotic treated, exhibit changes in electrophysiology over time. However, while both sample groups reported similar MDV and ζ-potential values at baseline, deviations from the baseline were observed sooner in the untreated, showing a significant change in both MDV and ζ-potential at 72 hours. After 48 hours, the untreated group had an approximate 15% ±  4.7% (mean ±  SEM) percentage change in ζ-potential from baseline, versus 4.2% ±  1.5% percentage change in the treated group. However, the difference between untreated and treated groups at 48 hours, as well as each individual group compared to baseline, was not significant.

These findings align with the urine sediment analysis reported by Ribeiro et al. [5]; no refrigerated samples reported changes in nitrite testing at 24 hours, compared to baseline results, and only one out of eighty samples exhibited a pH change (from 6 to 7) from baseline. Similarly, Tharsikayini et al. [8] investigated the effects of storage conditions on the pH, glucose and protein of healthy urine specimens (unpreserved at 25°C, unpreserved in refrigeration at 4°C and refrigerated with chemical preservatives) over a 72-hour testing period. Samples were spiked with either glucose or albumin at varying concentrations to observe changes in concentrations over time. The study reported that after 6 hours, the unpreserved samples at 25°C showed significant changes in pH, glucose and protein compared to baseline, performing the worst out of all test groups. The two chemical preservatives used (thymol and toluene) both caused significant changes compared to baseline in the three testing parameters after 6 hours, although to a lesser extent to the unpreserved sample stored at 25°C. The unpreserved refrigerated sample performed the best, maintaining sample pH for up to 48 hours, only reporting significant differences from baseline at 72 hours. Glucose and protein concentrations (both 20 mg/dL) did not show any statistically significant differences from baseline until 24 hours of storage. The reported performance by Tharsikayini et al. [8] of untreated urine in refrigeration was markedly better than the findings in our study, in terms of maintaining the sample pH. As the researchers spiked their samples with glucose and protein, direct comparisons to this study cannot be made. The glucose and protein levels were also monitored in this study but tested negative throughout the entire testing duration. This is expected in healthy individuals, where most proteins and glucose undergo renal filtration and reabsorption [38].

Turning to the electrical measurements of cells, the untreated group showed a noticeable decrease in MDV over time. This was also observable in the untreated ζ-potential, where by 96 hours the ζ-potential is notably more negative than at baseline, indicating an increase in the negative charge density. This may be due to the presence of bacterial colonies in untreated specimens at after 48 hours of storage, indicated by positive nitrite tests and microscopic observation. Bacterial urease breaks down urea into ammonia and carbon dioxide. This process results in the alkalization of urine and the formation of phosphate salts, which is supported by the rise in sample pH after 48 hours of refrigeration [41]. At higher pH values, there is a greater deprotonation of functional groups, which leads to an increased concentration of negative charges in the electrical double layer. As a result, the repulsive forces between particles become stronger, causing the ζ-potential to become more negative.

Although there are no studies on urinary ζ-potential in the literature, there have been several investigating the ζ-potential of bacteria and the influence of pH. Wilson et al. [42] investigated the difference in ζ-potential between capsulated and non-capsulated strains of *P. multocida*, as a function of pH (from pH 2–9). In both strains, an increase in electronegativity coincided with increasing pH. Furthermore, the capsulated strain exhibited a lower isoelectric point (pI), defined as the pH at which a molecule carries no net electrical charge, than the non-capsulated strain and possessed considerably lower ζ-potential throughout the entire pH range. For example, at pH 6, the ζ-potential of the capsulated and non-capsulated strains were approximately −24mV and −15mV, respectively. The bacterial capsule consists of densely packed complex polysaccharides, primarily consisting of hyaluronic acid, which provides improved hydrophilic and electronegative characteristics to the cell surface [42]. In cystitis, many bacteria are able to encapsulate themselves, increasing immune evasion, adherence to host tissue and increasing resistance to environmental stress [43,44]. The presence of bacterial capsules may be attributed to the variation in ζ-potential change over time in urine specimens, although no staining or electron microscopy was undertaken to verify the presence of capsules. Kłodzińska et al. [45] also reported a linear decrease in ζ-potential of *E. coli* and *S. aureus* as pH increased from 2 to 5. However, no significant changes in ζ-potential was observed in both bacterial strains after around pH 5; this was also observed by Wilson et al. [42], where the concomitant increase in electronegativity with rising pH was observed from pH 5.6 to 6. This may suggest that the increase in urinary ζ-potential with rising pH, observed in the untreated group of this study, may also no longer change significantly if the pH increased further. Interestingly, the ζ-potential of *E. coli* and *S. aureus* strains were reportedly less negative when killed using 2-propanol solution. Dead cells typically show different ζ-potential than their live counterparts [13], which could further affect the electrophysiology of urine specimens as bacterial colonies rapidly grow and urinary cellular constituents begin to degrade and undergo apoptosis. It is important to note that the linear increase in MDV and decrease in ζ-potential over time was not a trend followed in the 1% penicillin/streptomycin treated samples; the percentage change from baseline in both parameters increased over time, with a significant difference in ζ-potential at 96 hours.

An important aspect to consider is that urine specimens were treated with 1% penicillin/streptomycin, used as a standard antibiotic cocktail to prevent bacterial infection in cell culture. However, both penicillin and streptomycin are among the earliest antibiotics to be developed, discovered in 1928 and 1943 respectively, with their usage altered over time due to the emergence of antibiotic resistance [46,47]. As the most common cause of UTIs, accounting for approximately 70% to 95% of cases, uropathogenic *E. coli* strains have also developed resistance to these “legacy” antibiotics, which are now used clinically in more specialised or limited roles compared to newer drugs [40,48]. Nitrofurantoin or trimethoprim are recommended by NICE as the as the first-choice antibiotics to treat UTIs [40]. Therefore, future investigations should both optimise the treatment dosage but also explore the use of antibiotics relevant to urinary infections, to determine whether the bacteriostatic properties can be extended in refrigerated urine specimens.

Nevertheless, this study demonstrates the novel application of antibiotic preservation of urine specimens for electrophysiological analysis. While the data presented with penicillin/streptomycin treatment provides a solid foundation, using alternative antibiotics may overcome any possible resistance-associated constraints and potentially extend preservation time. The method of antibiotic treatment and refrigeration of urine specimens presented in this study has demonstrated a simple and effective approach to improving sample preservation. MDV and ζ-potential analysis, using the 3DEP cytometer and a commercial zetasizer, provide rapid, non-destructive, and label-free alternative methods for urine analysis, potentially supplementing traditional urinalysis in clinical settings.

## Supporting information

S1 File Experimental data for the manuscript. (DOCX)
