# Supplementary material for: Evaluating refrigeration and antibiotic treatment for maintaining urine electrophysiology
Source: PLoS One. 2025 Feb 25;20(2):e0319089. doi: 10.1371/journal.pone.0319089 (PMC11856264; doi:10.1371/journal.pone.0319089)
Supplement: S1 File — (DOCX) [file pone.0319089.s001.docx]

**Supplementary Information**

|  | | | **0h (Baseline)** | **24h** | **48h** | **72h** | **96h** |
| --- | --- | --- | --- | --- | --- | --- | --- |
| **Sample 1** | **Untreated** | Mean ζ-potential (mV) | -15.07 | -15.02 | -17.29 | -17.34 | -18.30 |
|  |  | ζ % from baseline | 0.00% | 0.33% | 14.73% | 15.03% | 21.40% |
|  |  | MDV | 0.3365 | 0.3070 | 0.2042 | 0.1214 | 0.1801 |
|  |  | MDV % from baseline | 0.00% | 8.76% | 39.31% | 63.91% | 46.48% |
|  |  | pH | 6 | 6 | 6.5 | 6.5 | 7 |
|  |  | Nitrite | Neg | Neg | Trace | Trace | Pos |
|  | **1% Penicillin / Streptomycin** | Mean ζ-potential (mV) | -14.50 | -14.10 | -13.94 | -11.20 | -10.20 |
|  |  | ζ % from baseline | 0.00% | 2.78% | 3.90% | 22.75% | 29.64% |
|  |  | MDV | 0.3170 | 0.3070 | 0.3507 | 0.3541 | 0.4022 |
|  |  | MDV % from baseline | 0.00% | 3.15% | 10.63% | 11.71% | 26.89% |
|  |  | pH | 6 | 6 | 6 | 6 | 6.5 |
|  |  | Nitrite | Neg | Neg | Neg | Trace | Trace |

|  | | | **0h (Baseline)** | **24h** | **48h** | **72h** | **96h** |
| --- | --- | --- | --- | --- | --- | --- | --- |
| **Sample 2** | **Untreated** | Mean ζ-potential (mV) | -8.99 | -9.06 | -8.83 | -12.46 | -12.65 |
|  |  | ζ % from baseline | 0.00% | 0.81% | 1.75% | 38.61% | 40.70% |
|  |  | MDV | 0.2566 | 0.2042 | 0.1117 | 0.4906 | 0.7080 |
|  |  | MDV % from baseline | 0.00% | 20.42% | 56.45% | 91.22% | 175.91% |
|  |  | pH | 5 | 5 | 5 | 6 | 6 |
|  |  | Nitrite | Neg | Neg | Trace | Pos | Pos |
|  | **1% Penicillin / Streptomycin** | Mean ζ-potential (mV) | -7.63 | -7.72 | -8.12 | -8.47 | -5.97 |
|  |  | ζ % from baseline | 0.00% | 1.23% | 6.41% | 11.07% | 21.69% |
|  |  | MDV | 0.2382 | 0.2641 | 0.2967 | 0.3126 | 0.4022 |
|  |  | MDV % from baseline | 0.00% | 10.85% | 24.56% | 31.20% | 68.83% |
|  |  | pH | 5 | 5 | 5 | 5 | 6 |
|  |  | Nitrite | Neg | Neg | Neg | Neg | Trace |

|  | | | **0h (Baseline)** | **24h** | **48h** | **72h** | **96h** |
| --- | --- | --- | --- | --- | --- | --- | --- |
| **Sample 3** | **Untreated** | Mean ζ-potential (mV) | -11.58 | -11.34 | -14.08 | -14.26 | -15.70 |
|  |  | ζ % from baseline | 0.00% | 2.06% | 21.57% | 23.13% | 35.59% |
|  |  | MDV | 0.3070 | 0.3395 | 0.3365 | 0.4968 | 0.5900 |
|  |  | MDV % from baseline | 0.00% | 10.58% | 9.60% | 61.82% | 92.19% |
|  |  | pH | 6 | 6 | 6.5 | 7 | 7 |
|  |  | Nitrite | Neg | Neg | Trace | Pos | Pos |
|  | **1% Penicillin / Streptomycin** | Mean ζ-potential (mV) | -11.46 | -11.43 | -11.43 | -11.41 | -13.11 |
|  |  | ζ % from baseline | 0.00% | 0.26% | 0.24% | 0.38% | 14.43% |
|  |  | MDV | 0.2958 | 0.3395 | 0.3365 | 0.3971 | 0.4412 |
|  |  | MDV % from baseline | 0.00% | 14.76% | 13.74% | 34.22% | 49.15% |
|  |  | pH | 6 | 6 | 6 | 6 | 6.5 |
|  |  | Nitrite | Neg | Neg | Neg | Neg | Trace |

|  | | | **0h (Baseline)** | **24h** | **48h** | **72h** | **96h** |
| --- | --- | --- | --- | --- | --- | --- | --- |
| **Sample 4** | **Untreated** | Mean ζ-potential (mV) | -9.94 | -9.05 | -7.77 | -15.55 | -15.80 |
|  |  | Zeta % from baseline | 0.00% | 9.04% | 21.85% | 56.33% | 58.84% |
|  |  | MDV | 0.0964 | 0.1111 | 0.0653 | 0.2418 | 0.2054 |
|  |  | MDV % from baseline | 0.00% | 15.21% | 32.27% | 150.82% | 112.98% |
|  |  | pH | 6.5 | 6.5 | 6.5 | 6.5 | 7 |
|  |  | Nitrite | Neg | Neg | Neg | Pos | Pos |
|  | **1% Penicillin / Streptomycin** | Mean ζ-potential (mV) | -10.19 | -10.54 | -10.84 | -12.18 | -12.91 |
|  |  | ζ % from baseline | 0.00% | 3.44% | 6.39% | 19.59% | 26.71% |
|  |  | MDV | 0.1712 | 0.1892 | 0.1041 | 0.3067 | 0.3971 |
|  |  | MDV % from baseline | 0.00% | 10.55% | 39.20% | 79.15% | 131.97% |
|  |  | pH | 6.5 | 6.5 | 6.5 | 6.5 | 7 |
|  |  | Nitrite | Neg | Neg | Neg | Pos | Pos |
